# Lipopolyplex for Therapeutic Gene Delivery and Its Application for the Treatment of Parkinson’s Disease

**DOI:** 10.3389/fnagi.2016.00068

**Published:** 2016-04-05

**Authors:** Wei Chen, Hui Li, Zhenguo Liu, Weien Yuan

**Affiliations:** ^1^Department of Neurology, Xinhua Hospital, Shanghai JiaoTong University School of MedicineShanghai, China; ^2^School of Pharmacy, Shanghai JiaoTong UniversityShanghai, China

**Keywords:** lipopolyplex, biological barriers, Parkinson’s disease, blood brain barrier

## Abstract

Lipopolyplex is a core-shell structure composed of nucleic acid, polycation and lipid. As a non-viral gene delivery vector, lipopolyplex combining the advantages of polyplex and lipoplex has shown superior colloidal stability, reduced cytotoxicity, extremely high gene transfection efficiency. Following intravenous administration, there are many strategies based on lipopolyplex to overcome the complex biological barriers in systemic gene delivery including condensation of nucleic acids into nanoparticles, long circulation, cell targeting, endosomal escape, release to cytoplasm and entry into cell nucleus. Parkinson’s disease (PD) is the second most common neurodegenerative disorder and severely influences the patients’ life quality. Current gene therapy clinical trials for PD employing viral vectors didn’t achieve satisfactory efficacy. However, lipopolyplex may become a promising alternative approach owing to its stability in blood, ability to cross the blood-brain barrier (BBB) and specific targeting to diseased brain cells.

## Introduction

### Gene Therapy and the Development of Lipopolyplex

Gene therapy is a therapeutic approach that aims to deliver exogenous genetic material (DNA/RNA) to a cell to correct a genetic defect or induce the expression of a specifically desired protein. It is extraordinarily powerful because the technique can be employed to correct genetic disorders or treat diseases with relatively well understood pathophysiology (Mustapa et al., 2007). However, the most crucial problem which needs to overcome in gene therapy is the development of an efficient, safe and convenient gene delivery vector.

**Figure 1:**
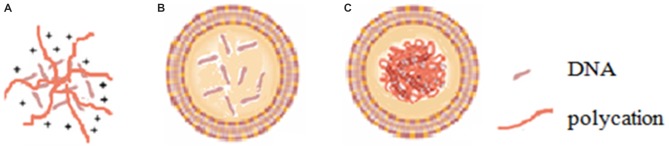
**Diagram of (A) polyplex, (B) lipoplex and (C) lipopolyplex**.

Viral vectors, especially adenoviral and retroviral systems, can provide high transfection efficiency and rapid expression of the foreign genetic material inserted into the viral genome and thus are currently the most widely used gene delivery vectors in the clinical stage. However, viral vectors have some inherent disadvantages including insertional mutagenesis, restriction to dividing cells, and relatively high immunogenicity (Somia and Verma, 2000), and severe problems have been observed during clinical trials of viral vectors (Marshall, 1999; Kang and Tisdale, 2004). On the other hand, non-viral vectors, mainly with cationic nature, typically involve the compaction of polyanionic nucleic acids with polycationic polymers (polyplexes; Figure 1A) such as polyethylenimine (PEI), dendrimers and peptides, or with cationic lipids (lipoplexes) (Miller, 1998; Davis, 2002; Figure 1B) through electrostatic interactions. The advantages of non-viral vectors over viral vectors include lower immunogenicity, easier scale-up manufacturing, more convenient modifications and higher packaging capacity. However, poor gene transfection efficiencies have limited their use to date.

Lipopolyplex (Figure 1C), a ternary complex of cationic liposome, polycation and DNA, has been developed as a second generation non-viral gene delivery vector following the first generation cationic liposome-DNA complex. Lipopolyplex combining the advantages of polyplex and lipoplex has shown superior colloidal stability, reduced cytotoxicity and extremely high gene transfection efficiency by virtue of the synergism of polycation and lipid (Li and Huang, 1997; Lampela et al., 2003; Lee et al., 2003; García et al., 2007; Ewe et al., 2014; Kurosaki et al., 2014). The first generation of lipopolyplex (LPD-I) consists of cationic lipid, protamine-based polycation and DNA (Gao and Huang, 1996). To overcome the cytotoxicity and improve biocompatibility of LPD-I, the second generation of lipopolyplex (LPD-II) was developed with the replacement of cationic lipid by anionic lipid (Lee and Huang, 1996). Munye et al. (2015) also reported a lipopolyplex formulation composed of liposome and peptide for gene delivery in the airway. The authors found that the peptide components and the liposome component of the lipopolyplex could have synergistic effects to promote cellular uptake as well as endosomal escape of its payloads.

### Biological Barriers in Gene Delivery

There are a variety of non-viral delivery strategies including physical methods, such as hydrodynamic injection (Liu et al., 1999; Stoll et al., 2001; Suda et al., 2008), particle bombardment (Belyantseva, 2009) and electroporation (Lee et al., 1992; Liu and Huang, 2002a,b,c), and chemical methods. In many cases, it is difficult to have access to some disease sites and local or tropical delivery of genetic materials usually is not efficient enough to achieve desired therapeutic efficacy. Therefore, intravenous administration will be needed. The following is the discussion about the biological barriers in systemic gene delivery following intravenous administration (Table 1).

**Table 1:** **Biological barriers to systemic gene delivery**.

Extracellular barriers	Intracellular barriers
Degradation by the nuclease in blood	Endosomal or lysosomal degradation
Clearance by kidney filtration	Movement to the target sites
Uptake by reticuloendothelial system	Translocation to the nucleus
Inability to target specific tissues or cells	
Movement inhibited by viscous mucus	
Inability to permeate cell membranes	

**Figure 2:**
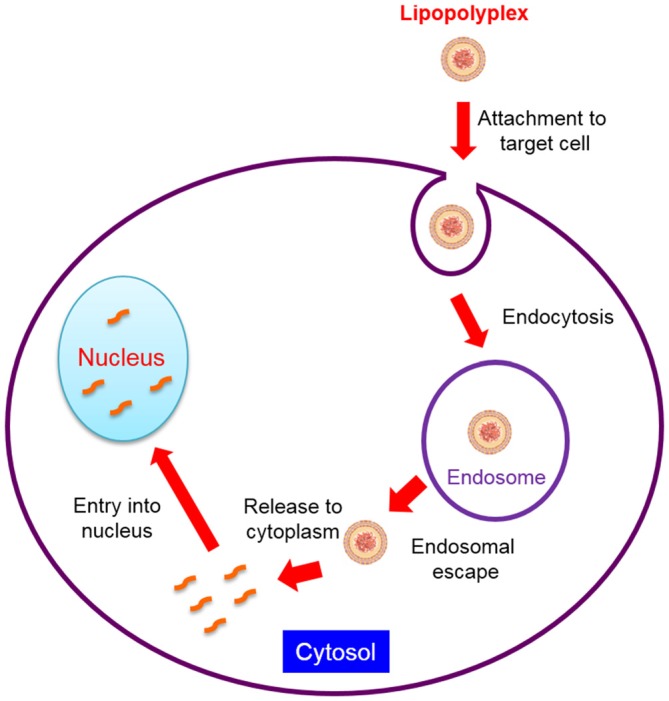
**Intracellular barriers for lipopolyplexes-mediated gene delivery**.

SiRNA, plasmid DNA (pDNA), miRNA and other un-modified oligonucleotides are unstable in the blood circulation and easily degraded by the nuclease. They are also prone to be rapidly cleared by kidney filtration after intravenous administration due to their relatively small size. In addition, to reach their target cells, they must evade uptake by reticuloendothelial system (RES), especially the Kupffer cells in the liver and the macrophages in the spleen. They also have to traverse from blood vessels and gain access to the target tissue if the blood cells and blood vessel cells are not the intended target. Although some tissues such as tumors, the RES and inflammatory sites have leaky blood vessels, the capillary vessel walls of most organs and tissues are not permeable to nucleic acids. Moreover, gene medicines are inhibited to move from extracellular matrix (ECM) to target cells due to the dense polysaccharides and fibrous proteins in the ECM (Zámecník et al., 2004). The intracellular barriers for lipopolyplexes-mediated gene delivery are further summarized in Figure 2. Nucleic acids are highly hydrophilic macromolecules with negative charges, which usually impede them to bind to and passively diffuse across lipophilic cell membranes. Even if they are uptaked by the endocytic pathway, the endosomal or lysosomal degradation is also a major issue. For siRNA-based therapeutics, they must escape from the endosome to reach the cellular cytoplasm where siRNA takes action. Furthermore, the cytosolic viscosity and dense organelles may prevent their movement towards target sites. In terms of pDNA, the nuclear envelope represents an extra and formidable barrier (Lam and Dean, 2010) and pDNA has to translocate to the nucleus for expression. Therefore, the translation of therapeutic nucleic acids into the clinical setting is largely dependent on the development of an appropriate delivery system which is able to overcome all the mentioned biological barriers.

### Strategies in Lipopolyplex-Based Systemic Gene Delivery

#### Condensation of Nucleic Acids into Nanoparticles

Nucleic acids as high molecular weight biomolecules are subjected to various environmental factors including pH and nucleases which can degrade them. Lipopolyplexes have been prepared by condensing nucleic acids into homogenous and tight particles (polyplex) with the aid of polycation and entrapping this polyplex within cationic (Liu and Huang, 2002a,b,c), anionic (Lee and Huang, 1996) or neutral (Ibáñez et al., 1996) liposomes. Poly-L-lysine (PLL), PEI, spermidine, spermine and protamine sulfate are the commonly used polycation in lipopolyplex systems. In lipopolyplex formulations, both Lys-rich and Arg-rich peptides can condense and protect nucleic acids effectively and lead to high transfection efficiency. However, lipopolyplexes prepared with His-containing cationic peptide sequences have relatively poor transfection efficiency, as these peptides make DNA difficult to escape from endosome and cannot condense and protect DNA adequately (Welser et al., 2013). PEI is available in different lengths, either branched (BPEI) or linear (LPEI), and PEI could be easily functionalized by functional group addition or substitution. PEI is also able to successfully condense nucleic acids into homogeneous spherical particles by virtue of the electrostatic interaction between the negatively charged nucleic acids and positively charged PEI. Studies showed LPEI with low molecular weight was the least cytotoxic and the most efficient carrier in nucleic acids transfection (Breunig et al., 2005). When the nitrogen residues are excess in comparison with the phosphate residues of DNA, PEI/DNA complexes are positively charged. The complexes could bind to cell surface with negative charge via electrostatic interaction and result in high efficient gene expression.

Protamine is a natural polycation which was found in the head of spermatozoa. Protamine plays a crucial role in condensing DNA in sperm and transferring it to the egg nucleus. The nuclear localizing property of protamine causes growing attention for using it for transfection. Protamine sulfate is a defined peptide system and its molecular weight ranges from 4 to 4.25 kDa. The most commonly used lipids in lipopolyplex system are cationic lipids made up of a cationic head group attached by a linker to a lipid hydrophobic moiety. They can be classified into various subgroups including the monocationic lipids, e.g., N[1-(2,3-dioleyloxy) propyl]-N,N,N-trimethylammonium chloride (DOTMA), 1,2-dioleyl-3-trimethylammonium-propane (DOTAP), N-(2-hydroxyethyl)-N,N-dimethyl-2,3-bis(tetradecyloxy-1-propanaminium bro-mide) (DMRIE), the polycationic lipids, e.g., DOSPA (2,3-dioleyloxy-N-[2(sperminecarboxamido)-ethyl]-N,N-dimethyl-1-propanaminium trifluoro acetate), and cationic cholesterol derivatives, e.g., bis-guanidium-tren-cholesterol (BGTC). Cationic lipids are usually employed with the so-called helper lipids such as 1,2-dioleoyl-sn-glycero-3-phosphoethanolamine (DOPE) or cholesterol to improve transfection efficiency (Hirsch-Lerner et al., 2005). To deliver DNA, LPD-I developed by Liu et al. (1999) is composed of protamine sulfate, DNA and DOTAP/cholesterol liposome. To deliver siRNA, it is condensed by protamine with the help of the high molecular weight calf thymus DNA and then entrapped into pegylated liposome for surface protection (Li et al., 2008a,b). In this lipopolyplex system, calf thymus DNA is used to increase the negative charge density of siRNA and facilitate the formation of compact polyplex nanoparticles in the core.

#### Long Circulation

Intravenous administration of positively charged polyplexes, lipoplexes or lipopolyplexes may lead to significant toxicity and low transfection efficiency owing to binding to plasma proteins or blood cells nonspecifically via electrostatic interaction, or activating the complement system. For example, 60% of the delivered pDNA by cationic liposome following intravenous injection in mice accumulated in the liver. However, the level of gene expression per microgram of DNA uptaked in the liver was 1000 times lower than that in the lung, which may be due to that DNA was rapidly degraded after phagocytosis by the Kuffer cells (Kabanov, 1999). The reason why lipoplex tends to be taken up by RES is that opsonins like IgG, IgM, complement C3 or fivronectins binding to the bare surface of lipoplex can attract phagocytic cells.

Two ways to deal with this problem are neutralizing the positive charges with anionic membrane components and shielding the surface charge by PEGylation, respectively. To overcome the cytotoxicity and enhance efficiency of LPD-I, LPD-II was first complexed to polylysine at a ratio of 1:0.75 (w/w) and then entrapped into folate-targeted pH-sensitive anionic liposomes (Lee and Huang, 1996). The difference between LPD-II and LPD-I is that anionic lipids instead of cationic lipids are used. An anionic lipopolyplex system for the delivery of synthetic miR-29b mimic molecules with the membrane component of linoleic acid was developed recently (Huang et al., 2013). Kurosaki et al. (2009) prepared lipopolyplexes composed of PEI, DOTMA, N-lauroylsarcosine (LS), and pDNA. Studies showed that lipopolyplexes containing LS which decreased the high zeta potential showed little aggregation with erythrocytes and low cytotoxicity. On the other hand, stealth liposome with surface grafted hydrophilic molecules such as polyethylene glycol (PEG) is commonly used to reduce the surface positive charge, to decrease the particle-particle interaction and prevent aggregation, and to protect particles from uptake by the RES system. PEGylation can create a hydrophilic cloud around the particle surface resulting in steric hindrance between the delivery carriers and the opsonins and thus prolong the circulation time in blood and hence improve systemic gene delivery (Muzykantov and Torchilin, 2003). PEGylation of liposomes can increase the half-life of liposomes in blood to 6–10 h in mice and to 40 h in human (Woodle, 1993). Although PEGylation greatly improved the stability of stealth liposome *in vivo*, in many cases cell transfection efficiency was dramatically reduced. This phenomenon is attributed to numerous factors, such as particle positive charge masking (Erbacher et al., 1999), increase of steric hindrance of targeting ligands (Ogris et al., 2001), interference with the interactions with cellular membranes and endosomal membranes (Song et al., 2002), impeding of intracellular pDNA trafficking (Keller et al., 2003). In many cases the targeting ligands coated on the surface of the stabilized particles did not enhance the transfection efficiencies (Rudolph et al., 2002). One way to solve the emergent problem was pH-sensitive PEGylation of lipopolyplex-based vector. With pH-sensitive PEGylation, the PEG shield was expected to be removable in intracellular endosomes. For example, Nie et al. (2011) developed pyridylhydrazone-based PEGylated lipopolyplexes for pH-reversible shielding. The lipopolyplex consisted of DNA condensed with PEI, DOPE and [u-2-pyridyldithio poly (ethylene) glycola-(butyraldehyde) (N1-cholesteryloxycarbonyl-1, 2-diaminoethane amidocarboxy) pyridyl hydrazone] (OPSS-PEG-HZN-Chol) which was an endosomal pH-cleavable reagent. Studies showed that transfection with plasmids encoding for luciferase or EGFP was 40 times higher in gene expression with the reversibly PEGylated lipopolyplexes compared to the stably PEGylated ones (Nie et al., 2011).

Furthermore, it is essential to mention that the lipopolyplex formulation contained up to 10 mol% of PEGylation (Li et al., 2008b), while lipid bilayer can maximally tolerate only 5–6 mol%. The reason may be that the detergent-like activity of the PEG-phospholipid conjugate can lyse liposomes at high concentrations. However, the charge-charge interaction between the shell and core in the lipopolyplex system stabilizes it and make it tolerate a high amount of the conjugate. The high degree of PEGylation may be the reason why the lipopolyplex is not uptaked by the spleen and liver to a significant degree (Li et al., 2008b).

#### Cell Targeting

Following condensing nucleic acids into nanoparticles and prolonging blood circulation time, the next challenge for gene delivery vectors is to target cells of interest. Cell targeting can be divided into two categories, passive targeting and active targeting. At the site of solid tumor, the leaky and discontinuous neovasculature together with the lack of lymphatic drainage lead to the accumulation of macromolecules and colloidal nanoparticles with diameters ranging from 100 nm to 200 nm, a phenomenon called “enhanced permeability and retention” (EPR) effect (Fang et al., 2011). Cell targeting taking advantages of EPR effect belongs to passive targeting. However, emphasis must be laid on that the degree of leakiness of tumor endothelium differs among different tumors (Hashizume et al., 2000) and not all human tumors are equally leaky (Konno et al., 1983, 1984; Maki et al., 1985; Seymour et al., 1998). Li et al. (2008a) developed a lipopolyplex vector composed of protamine, siRNA, calf thymus DNA and PEGylated liposome. Taking advantage of the EPR effect, a high dose (60–80% intravenously administered dose per gram of tissue) of accumulation was showed in the H460 lung cancer xenograft model.

Active targeting involves the covalent attachment of targeting ligands, such as peptides, proteins, antibodies, small molecules and nucleic acid aptamers, to the surface of a delivery system, which will specifically interact with receptors over-expressed on the surface of target cells and lead to high transfection efficiency (Ogris and Wagner, 2002). For example, the incorporation of folate ligands into the lipopolyplexes enhanced gene transfection efficiency (Lee and Huang, 1996). Li et al. (2008a) prepared a PEGylated lipopolyplex formulation with anisamide as the targeting ligand. The formulation efficiently delivered siRNA to the tumor cells expressing sigma receptor and almost completely silenced the target gene following three daily intravenous administrations. ErbB2 is a member of the EGFR family and over-expressed in breast and ovarian cancer cells (Lee et al., 1995). A lipopolyplex with a single-chain antibody fragment (ScFv) against ErbB2 transferred pDNA to over-expressing ErbB2 cell lines and achieved higher expression of the luciferase reporter gene in the ErbB2 positive cells than the ErbB2 negative cells (Li et al., 2001). Integrins are heterodimeric transmembrane proteins comprised of an a- and b-subunit that define ligand specificity. They play important roles in mediating cell-substratum and cell-cell interactions (Hynes, 1987). The arginine-glycine-aspartic acid (RGD) motif is a classic targeting ligand for integrin. Scott et al. (2001) developed a lipopolyplex with the targeting ligand RGD. Results showed that the integrin-targeting peptide was capable of binding to the RGD motif located on the apical surface of a polarized human bronchial epithelial cell line (16HBE) and enhance the luciferase gene transfer efficiency.

#### Endosomal Escape

After lipopolyplexes gain access to the target cells and are internalized via the receptor-mediated endocytic pathway, they are entrapped into the endosome where nucleic acids undergo degradation. Lipopolyplexes can escape from endosome combining the advantages of both polyplexes and lipoplexes through ion-pair formation and proton sponge effect.

##### Ion-pair formation

Cullis’ group suggested the mechanism of the destabilization of endosomal membrane by cationic lipids (Hafez et al., 2001). Following endocytosis, the cationic lipids and the anionic lipids in the endosome membrane could form ion-pairs which make the endosomal membrane destabilized by virtue of excluding the surface bound water (Xu and Szoka, 1996). The electrical interaction between cationic lipids and anionic lipids could further promote the formation of the inverted hexagonal (HII) phase and disrupt the endosomal membrane. Lipids with smaller and/or less hydrophilic head groups and bulky acyl or alkyl chains could facilitate the formation of HII phase (Xu and Szoka, 1996). In addition, HII phase is reported to be an intermediate structure formed during the fusion of two lipid bilayers with each other (Hafez and Cullis, 2001; Ewert et al., 2005). Both bilayers are destabilized in the process of fusion. DOPE is a helper lipid for destabilizing endosomal membrane and contained in many lipopolyplex formulations. The fusogenic functionality of DOPE is due to its ability to form the HII phase. DOPE with a large hydrophobic hydrocarbon area and a small hydrophilic headgroup favors the formation of a non-bilayer structure with a cone shape which leads to the destabilization of endosomal membranes and improves gene transfer efficiency (Farhood et al., 1995; Fasbender et al., 1997; Hafez and Cullis, 2001).

##### Proton sponge effect

Nucleic acids are condensed by cationic polymers in the core of lipopolyplexes. In the endosome with typically lower pH, the cationic polymer behaves as a sponge which leads to an influx of protons due to protonation of the primary, secondary or tertiary amine groups. The counter chloride ions are pumped into the endosome along with protons to maintain charge neutrality, which result in high osmotic pressure and subsequently influx of water and eventually rupture of the endosomal membrane (Cho et al., 2003).

#### Release to Cytoplasm

siRNA must be released from siRNA complex to the cytoplasm so that the free siRNA could get access to the RNA-induced silencing complex (RISC) for gene silencing. Strategies for efficient release of siRNA from nanoparticles include pH-sensitive detergent (Asokan and Cho, 2002), acid-labile cross-linkers (Guo and Szoka, 2003), enzyme active linker, redox-responsive disulfide cross-linker and so forth (Musacchio et al., 2010; Son et al., 2012). For example, redox-sensitive controlled release of siRNA is a commonly used strategy. The ECM and intracellular cytosol are highly oxidizing and reducing, respectively. In the cytosol, glutathione (GSH) exists in both reduced (GSH) and oxidized (disulfide) states and its concentration is about 1000 times higher which leads to a high redox potential gradient between the intracellular and ECM (Ouyang et al., 2009). Therefore, disulfide bonds will be reduced in the cytosol leading to high gene delivery efficiency.

#### Entry into Nucleus

Unlike siRNA, pDNA has to be transported from cytoplasm to nucleus which occurs during cell division along with breaking down of the nuclear envelope or via pores in the nuclear membrane. There is evidence showing that the nuclear membrane pores could act as a barrier for large particles. Small size DNA is passively diffused into the nucleus while large DNA complex is energy-dependently transported via the nuclear pore complex (Kreiss et al., 1999; Ludtke et al., 1999). Import efficiency via nuclear pore can be enhanced by the employment of nuclear localization signal (NLS) peptides. For example, Wiseman et al. (2005) prepared a lipopolyplex system with a synthetic peptide which is based on the amino terminal region of the polyoma virus VP1 protein. This region has overlapping yet functionally distinct motifs for nuclear localization and DNA binding which provides an easy approach of incorporating a NLS peptide into a gene delivery system through electrostatic interaction with DNA. Results showed that a lipopolyplex consisting of the VP1 peptide promoted gene delivery because VP1 increased the amount of plasmid associated with the nucleus (Wiseman et al., 2005).

### Introduction of Parkinson’s Disease

Parkinson’s disease (PD) is the second most common neurodegenerative disorder which is dependent on age. The normal onset age of PD is about 65 years old and the early-onset age shown in some cases is around 45 in a small portion of the population affected with PD (Tanner et al., 1997; McNaught and Olanow, 2006; Rao et al., 2006). The number of PD patients is about 1% of the population and estimated to increase from 4.1 million in 2005 to 8.7 million in 2030 (Robinson, 2008). PD costs approximately €14 billion in 2010 in Europe (Gustavsson et al., 2011) and has significant influences on the life quality of patients and their families. The symptoms of PD differ among patients and may be involved in loss of spontaneous movement, resting tremor, bradykinesia, cogwheel rigidity, postural instability, decreased clarity and volume in speech, and less legible handwriting (Savitt et al., 2006; Tolosa et al., 2006; Jankovic, 2008; Pahwa and Lyons, 2010). The diagnosis of PD patients is mainly based on a comprehensive physical and neurological examination and patients’ medical history. Parkinson (2002) described PD in 1817 at the first time and report in 1893 showed that it was the degeneration of dopaminergic neurons in the substantia nigra (SN) pars compacta that resulted in the behavioral complications of PD (D’Amelio et al., 2009). Along with the progress of the disease, other brain regions are involved, such as the amygdale, cingulate gyrus and higher cortical regions leading to psychiatric demonstrations and dementia in PD patients. Pathologically, Lewy bodies formed by unusual aggregates of the protein α-synuclein in the dopaminergic neurons are considered as the hallmark of PD (Harraz et al., 2011). PD primarily manifests in a sporadic fashion (Cookson and Bandmann, 2010). Hereditary factors have a relatively small influence because merely about 10% of patients are involved in genetic links which mainly lead to early-onset PD (Bekris et al., 2010). To date, mutations of four genes are certified to result in autosomal recessive PD, consisting of PINK1 (PARK6; Valente et al., 2004), parkin (PARK2; Kitada et al., 1998), ATP13A2 (PARK9; Ramirez et al., 2006) and DJ-1(PARK7; Bonifati et al., 2003). Deficiency of the function of any single gene is capable of causing the degeneration of dopaminergic neurons and symptoms of PD. Other possible factors comprise medications, environmental toxins and viruses which all lead to the increase of oxidative stress (Di Monte et al., 2002; Jenner and Olanow, 2006). Oxidative stress can result in the yield of free radicals that causes cell death in dopamine neurons (Baldessarini and Tarazi, 1996; Naoi and Maruyama, 1999).

Pharmacotherapy is often employed for the treatment of mild PD. Levodopa is commonly used for PD and leads to sustained benefit, while long time use can result in response fluctuations and dyskinesias. Synthetic dopamine receptor agonists can cause non-physiological stimulation of dopamine receptor. However, they can in turn induce serious disorders of impulse control apart from other adverse effects such as hallucinations, excessive daytime somnolence and postural hypotension (Weintraub et al., 2010). Along with the advancement of PD, non-motor problems such as cognitive and behavioral disability arise, which have a more significant influence on patients’ life quality than motor dysfunction (Martinez-Martin et al., 2011). The majority of these problems are hardly resolved by dopaminergic therapy.

Gene therapy is an innovative approach for the treatment of PD. During the past 10 years, nine PD clinical trials (Table 2) applying gene therapy approach have been carried out and completed. All of them have employed adeno-associated virus (AAV) or lentivirus as gene delivery vectors focused on symptomatic or disease-modifying impacts. The symptomatic means were aimed at either the normalization of basal ganglia circuitry via changing the neuronal phenotype or the increase of dopamine yield via transferring genes associated with the synthesis of neurotransmitter (Feng and Maguire-Zeiss, 2010). As for disease-modifying means, several clinical trials have been carried out by delivering a gene that expresses a neurotrophic factor with the aim of increasing dopaminergic nerve terminals. Owing to the disability of the viral vectors across the blood-brain barrier (BBB), all clinical trials for PD to date have infused the vector into the target sites in brain by virtue of a craniotomy. Although each clinical trial was started with considerable optimism, none of them has yielded sufficiently significant efficacy or proceeded with regulatory approval.

**Table 2:** **Summary of gene therapy clinical programs for Parkinson’s disease (Bartus et al., 2014)**.

Treatment (approach)	Trial design	Year began	Subject # dosed	Highest total dose(vg)	Target(S)	Largest volume (μl)/site	Safety results	Efficacy outcomes
AAV2/GAD	Ph1-uncontrolled^a^	2003	12	1 × 10^12^	Subthal Nuc (unilat)	50	Acceptable	Advanced to Ph2
	Ph2-double-blind^b^	2008	22/16*	1 × 10^12^	Subthal Kuc (Bilat)	35	Acceptable	Mixed results; program suspended
AAV2/AADC	Ph1-uncontrolled^c^	2004	10	0.3 × 10^12^	Putamen (Bilat)	50	Acceptable	Program suspended; revised Ph1 recently announced
AAV2/AADC	Ph 1 -uncontrolled^d^	2007	6	0.3 × 10^12^	Putamen (Bilat)	50	Acceptable	No further testing; revised Ph1 recently announced by USA group
AAV2/	Ph1-uncontrolled^e^	2005	12	0.54 × 10^12^	Putamen (Bilat)	5(10)**	Acceptable	Advanced to Ph2
NRTN	Ph2A-double-blind^f^	2006	38	0.54 × 10^12^	Putamen (Bilat)	5(10)**	Acceptable	Mixed results; revised Ph1 designed
	Ph1-uncontrolled^g^	2009	6	2.4 × 10^12^	Put + SN (Bilat)	50	Acceptable	Advanced to Ph2
	Ph2B-double-blind^h^	2010	24	2.4 × 10^12^	Put + SN (Bilat)	50	Acceptable	Program suspended
LENTI/AADC-	Ph 1/2-uncontrolled^#^	2008	15	Lentivirus dosing is not comparable to that of AAV^##^	Putamen (Bilat)		Acceptable	Program suspended; additional work to optimize vector ongoing
AAV2/GDNF	Ph1-uncontrolled^k^	2013	Ongoing	0.7 × 10^12^	Putamen (Bilat)	150	N/A	N/A
Synopsis	Total of seven phase 1 and three phase 2 trials	2003–2013	>139	Tested up to 1 × 10^12^ vg AAV	Targets have included subthalamic nucleus, putamen and SN	50 μl (most common); 150 μl (largest)	No safety issues or serious side effects noted	Efficacy outcomes generally disappointing

AAV, adeno-associated virus; SN, substantia nigra. ^a^Kaplitt et al. (2007); ^b^LeWitt et al. (2011); ^c^Christine et al. (2009); ^d^Muramatsu et al. (2010); ^e^Marks et al. (2008); ^f^Marks et al. (2010); ^g^Bartus (2013); ^h^Bartus et al. (2013); Palfi et al. (2014); and ^k^Lonser (2009). *Twenty-two subjects were dosed but six were eliminated from efficacy analysis due to mistargetirvg of cannula. **Two 5 μl volumes infused via single needle tract −4 mm apart. ^#^Described as a Phase 1/2 trial, this open label (uncontrolled) study does not differ substantially from many dose-escalation Phase 1 safety studies that include secondary efficacy endpoints; thus, the distinction appears to be more a semantic preference than a reflection of a substantial difference in study design. ^##^A fivefold dose range was tested involving three dose levels (1.9 × 10^7^ transducing units (TU); 4.0× 10^7^ TU; 1.0× 10^8^ TU).

A few non-viral approaches of gene delivery for PD treatment are being tested in preclinical cases. For instance, direct injection applying electroporation or a gene gun is possible to transfer genes efficiently (Coune et al., 2012). Apart from direct injection, intranasal administration of genetic materials may be devoid of the technical difficulty of making gene therapy overcoming the BBB and have access to the central nervous system (CNS; Lao et al., 2013; Waszczak et al., 2013).

### Outlook of Lipopolyplex for the Treatment of Parkinson’s Disease

The current gene therapy approach of PD in clinical trials is utilizing AAV or lentivirus as gene delivery vectors. Generally, AAV vectors have to be administered at intervals and pre-existing immunity to AAV is present in approximately 90% of population (Chirmule et al., 1999). It is reported that both retrovirus and AAV permanently or randomly insert into the host genome causing gene mutation (Miller et al., 2002; Laufs et al., 2003). Owing to their disability of crossing the BBB, AAV and retrovirus need to be administered by virtue of transcranial injection which considerably reduces patient’s compliance. In addition, because the viruses diffuse limitedly in the brain, the most significant expression region of the delivered gene is usually restricted to the injection site. With so many deficiencies, none of the gene therapy clinical trials of PD applying virus vectors has yet found a clear path to regulatory approval.

**Figure 3:**
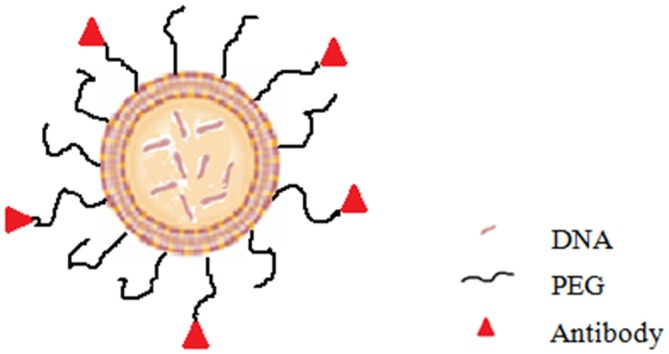
**Diagram of immunoliposome**.

Current problems of PD gene therapy approaches can be resolved by the development of an intravenous delivery approach which require that the formulation of therapeutic gene was able to cross the BBB and target the specific cell in the brain. As mentioned above, the vector of lipopolyplex have the potential to meet this requirement. Compared with viral vectors, the delivery system of lipopolyplex has many significant advantages such as higher gene-packaging capability, lower immunogenicity, more convenient modification and easier scale-up manufacture. More essentially, lipopolyplex is able to have long circulation time in the blood, cross the BBB and enter the target cells in brain. Some researchers developed the immunoliposomes (antibody-directed liposomes; Figure 3) for gene therapy of PD. In the immunoliposomes, nucleic acids can be delivered via liposomes that are coated with PEG and further modified with antibody for targeting to the CNS (Huwyler et al., 1996; Pardridge, 2003). For example, the “Trojan Horse Liposome” (THL) is such a immunoliposome which is a promising alternative for gene delivery to the CNS (Shi and Pardridge, 2000; Shi et al., 2001a,b; Zhang et al., 2003, 2004). In the THLs, DNA is encapsulated in the internal cavity of the liposome, which protects DNA from nuclease degeneration. The THL is prepared by lipids comprising PEG that prolong the circulation time in the blood (Gabizon and Papahadjopoulos, 1988; Papahadjopoulos et al., 1991). About 1–2% of the PEG residues are conjugated to the peptidomimetic monoclonal antibodies (MAb) which can specifically target the receptors, such as insulin and transferrin receptors, distributed on both the BBB and the brain cellular membranes, respectively (Shi and Pardridge, 2000; Shi et al., 2001a,b; Zhang et al., 2003, 2004). The THL acts as a molecular Trojan horse, because these MAbs on the surface of the THLs can help to mediate the receptor-mediated transcytosis across the BBB, endocytosis into the neurons behind the BBB and then transport to the nuclear compartment (Boado, 2007). Studies showed that in the 6-hydroxydopamine rat model of PD, transferrin receptor MAb-targeted immunoliposomes loading a tyrosine hydroxylase (TH) expression plasmid completely normalized the striatal TH activity (Pardridge, 2005). According to their formulation, the mentioned immunoliposome is a kind of lipoplex. Compared with lipoplex, lipopolyplex can have higher gene transfection efficiency. Although to date no lipopolyplex system for PD gene therapy has been reported, it is expected to be an extremely promising delivery vector based on the above analysis.

## Author Contributions

WC participated in its design, searched databases, extracted and assessed studies and helped to draft the manuscript. HL, ZL and WY participated in the conceptualization and design of data extraction and analysis, wrote and revised the manuscript. All authors read and approved the final manuscript.

## Conflict of Interest Statement

The authors declare that the research was conducted in the absence of any commercial or financial relationships that could be construed as a potential conflict of interest.
